# Heel pressure ulcer, prevention and predictors during the care delivery chain – when and where to take action? A descriptive and explorative study

**DOI:** 10.1186/s13049-016-0326-0

**Published:** 2016-11-14

**Authors:** Åsa Muntlin Athlin, Maria Engström, Lena Gunningberg, Carina Bååth

**Affiliations:** 1 Department of Emergency Care and Internal Medicine, Uppsala University Hospital, Entrance 40, 751 85 Uppsala, Sweden; 2 Department of Medical Sciences, Uppsala University, Uppsala, Sweden; 3 Department of Public Health and Caring Sciences, Uppsala University, Uppsala, Sweden; 4 School of Nursing, University of Adelaide, Adelaide, Australia; 5 Faculty of Health and Occupational Studies, Department of Health and Caring Sciences, University of Gävle, Gävle, Sweden; 6 Nursing Department, School of Medicine and Health, Lishui University, Lishui, China; 7 Quality Department, Uppsala University Hospital, Uppsala, Sweden; 8 Faculty of Health, Sciences and Technology, Department of Health Sciences, Karlstad University, Karlstad, Sweden; 9 County Council of Värmland, Karlstad, Sweden

**Keywords:** Acute care, Ambulance, Emergency department, Nursing intervention, Pressure ulcer, Prevention, Quality indicator

## Abstract

**Background:**

Hazardous healthcare settings, for example acute care, need to focus more on preventing adverse events and preventive actions across the care delivery chain (i.e pre-hospital and emergency care, and further at the hospital ward) should be more studied. Pressure ulcer prevalence is still at unreasonably high levels, causing increased healthcare costs and suffering for patients. Recent biomedical research reveals that the first signs of cell damage could arise within minutes. However, few studies have investigated optimal pressure ulcer prevention in the initial stage of the care process, e.g. in the ambulance care or at the emergency department. The aim of the study was to describe heel pressure ulcer prevalence and nursing actions in relation to pressure ulcer prevention during the care delivery chain, for older patients with neurological symptoms or reduced general condition. Another aim was to investigate early predictors for the development of heel pressure ulcer during the care delivery chain.

**Methods:**

Existing data collected from a multi-centre randomized controlled trial investigating the effect of using a heel prevention boot to reduce the incidence of heel pressure ulcer across the care delivery chain was used. Totally 183 patients participated. The settings for the study were five ambulance stations, two emergency departments and 16 wards at two hospitals in Sweden.

**Results:**

A total of 39 individual patients (21 %) developed heel pressure ulcer at different stages across the care delivery chain. Findings revealed that 47–64 % of the patients were assessed as being at risk for developing heel pressure ulcer. Preventive action was taken. However, all patients who developed pressure ulcer during the care delivery chain did not receive adequate pressure ulcer prevention actions during their hospital stay.

**Discussion and Conclusions:**

In the ambulance and at the emergency department, skin inspection seems to be appropriate for preventing pressure ulcer. However, carrying out risk assessment with a validated instrument is of significant importance at the ward level. This would also be an appropriate level of resource use. Context-specific actions for pressure ulcer prevention should be incorporated into the care of the patient from the very beginning of the care delivery chain.

**Trial registration:**

ISRCTN85296908.

**Electronic supplementary material:**

The online version of this article (doi:10.1186/s13049-016-0326-0) contains supplementary material, which is available to authorized users.

## Background

Healthcare economy and effectiveness are highlighted in many healthcare systems around the world, leading to more focus on the health professionals and on how to maximize their work efficiency and increase their use of best evidence practice in patient care [1, 2]. To achieve these goals, a great deal of focus is placed on the prevention of adverse events and how to use resources most effectively [2–4]. The failure to provide a high quality of care and prevent adverse events can negatively affect the economy and effectiveness of healthcare [5, 6].

The main mission of pre-hospital and emergency care is to treat and manage patients in the acute phase of illness and injury, often followed by hospital-based acute care. According to the Institute of Medicine in the United States, the acute care field is the most hazardous healthcare setting [7]. Complex acute care processes, often involving several health professionals and patients in a vulnerable situation, are challenging patient safety issues. Due to this, more focus is needed on preventing adverse events and identifying what preventive actions can be taken during the care delivery chain (i.e pre-hospital and emergency care level, and further at the hospital ward).

Pressure ulcer prevalence is used worldwide as a quality indicator, providing a benchmark to evaluate care in various settings [8, 9]. Studies show prevalence in hospital settings ranging between 0 and 46 % [8–10]. To identify patients at risk for pressure ulcer is a central component of clinical practice [10]. International guidelines specify that risk assessments for pressure ulcer development should be conducted within a maximum of eight hours after admission to hospital [10] and the mainstay of pressure ulcer prevention is pressure relief. However, the challenge is to implement evidence-based pressure ulcer prevention in clinical practice [11], and specifically in the initial stage of the care delivery chain.

Few studies have investigated optimal pressure ulcer prevention in emergency care settings; even fewer focus on pressure ulcer prevention in the pre-hospital care, for example ambulance care. Previous research in these areas has mainly considered pressure ulcer prevention in relation to stretchers, to support the body in the case of spine injury, and trauma patients [12]. Pressure ulcer prevention in the ambulance care could be of importance to many patients, as the transportation time can vary from minutes to hours, depending on the geographical circumstances. This immobilized state on an ambulance stretcher can be negative in cases of long transportation time. Further on, emergency department (ED) crowding is a problem around the world, leading to increased waiting times as well as safety and quality considerations [13–15]. A high number of these patients are in a fragile situation, with high age and suffering from multi-diseases leading to a complex situation. Also, recent biomedical research reveals that a direct deformation of tissue results from high strain, and is a process that leads to the first signs of cell damage within minutes [16]. Subsequently, patients in the ambulance or at the ED are exposed to high pressure ulcer risk [17–19].

This study is part of a larger research project investigating heel pressure ulcer prevention interventions across the care delivery chain. The first study, a randomized controlled trial (RCT), tested a heel suspension device boot applied in the ambulance, where patients were followed until discharged from the ward. Findings showed that patients in the intervention group had fewer heel pressure ulcers compared to patients in the control group, across the care delivery chain [20]. Noteworthy is that both groups of patients developed heel pressure ulcers across the care delivery chain. It is important to further study how to prevent heel pressure ulcer from the very beginning of the care delivery chain, to know where to take action and how to use healthcare resources effectively. Therefore, another study from the larger research project was carried out. The aim was to describe heel pressure ulcer prevalence and nursing actions in relation to pressure ulcer prevention during the care delivery chain (i.e pre-hospital and emergency care level, and further at the hospital ward), for older patients with neurological symptoms or reduced general condition. Another aim was to investigate early predictors for the development of heel pressure ulcer during the care delivery chain.

## Methods

### Design

A prospective, descriptive and explorative design was used, using data from a Swedish multi-centre RCT [20].

### Setting and sample

A total of five ambulance stations, two EDs, and 16 wards at two hospitals were included in the original RCT. Two county councils, were involved. Both county councils are widespread, which could generate ambulance transports of longer than 60 min. Since 2000, it is mandatory to have at least one registered nurse (RN) in each Swedish ambulance. Each of the EDs has more than 50,000 patient visits annually. Structured risk assessments for pressure ulcer were not routinely conducted in the ambulances and the EDs included in the study. On hospital level, the goal was to perform risk assessment within 24 h for all patients older than 65 years, but many Swedish hospitals are struggling to achieve this goal [21].

**Fig. 1:**
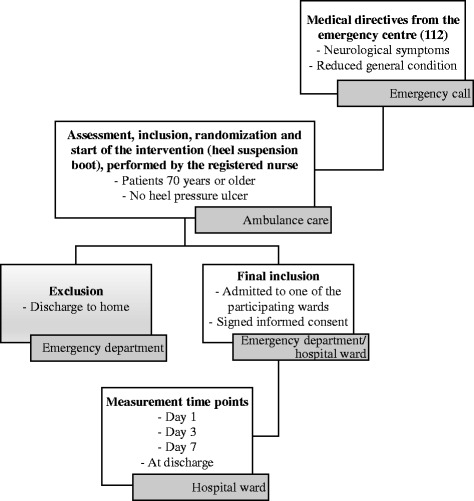
Procedure for inclusion and exclusion of target group

The target group was older patients (70+) with “neurological symptoms” or “reduced general condition”, according to medical directives from the emergency call centre, without a heel pressure ulcer (Fig. 1). The decision to approach such a broad target group was based on the facts that patients with vague symptoms and no/little abnormal vital signs are often lower prioritized in the ED leading to longer waiting-times. Also, diagnoses are seldom set at the start of the ambulance transportation and it was important to include the ambulance setting. Patients in need of life-threatening support and patients discharged from the ED were excluded. In addition, patients who were not able to sign informed consent or did not have a family-member to sign were excluded.

### Data collection

Before study start, all included settings received oral and written information about the study. Data were retrieved from study-specific protocols and the following was documented: ambulance) skin inspections of patients’ heels and measuring of vital signs; ED) skin inspections of patients’ heels, measuring of vital signs and risk assessment for pressure ulcers; ward) skin inspections of patients’ heels, measuring of vital signs and risk assessment for pressure ulcers. In addition, patients received care according to the pressure ulcer prevention routines of that ward. Patients in the intervention group also received a heel suspension device boot [20] (Additional file 1). Data collection was mainly carried out by the nurses and as a quality control study nurses were used. The study nurses performed additional skin inspections, risk assessments and collected the informed consents. The included hospitals used national guidelines for pressure ulcer prevention [10, 22]. All study nurses received specific education [23] and practical training and were supervised by the research team. Heel pressure ulcer category (Categories 1–4) was assessed according to an international classification system [10]. Risk assessment was performed using the validated Modified Norton Scale (MNS) [24].

### Data analysis

Descriptive statistics were used, and findings are presented as frequency, median (Md), means, mean rank, proportion, standard deviation (SD), quartile (Q) and range. To compare patients who developed heel pressure ulcer with those who did not, Chi-2 test, Mann-Whitney U-test and t-test were used (Additional file 1). Significance level was set to *p* < 0.05. The IBM Statistical Package for the Social Sciences (SPSS) version 19 was used for all analyses. Missing data were not replaced, thus for some analyses the number of participants do not add up to n.

## Results

A total of 183 patients were included, 63 male and 114 female. Mean age was 86.3 years (SD 7.2) (Md 86 years; Q1 81 years; Q3 91 years; range 70–100). Median ambulance transportation time was 25 min, with nine patients (5%) having an ambulance transportation time of 60–100 min (Q1 12 min; Q3 42.5 min). Total hospital stay varied from 0 to 74 days (Md 6 days; Q1 3 days; Q3 9 days), with four patients staying in the hospital for less than 24 h.

### Heel pressure ulcer across the entire care delivery chain

Skin inspections were performed at different time points across the care delivery chain for the majority of the included patients (ED *n* = 169 (92 %); ward day 1 *n* = 168 (92 %); ward day 3 *n* = 100 (87 %) and; ward day 7 *n* = 49 (100 %)). Heel pressure ulcer developed at different stages: 15 patients (9 %) were identified with heel pressure ulcers at the ED, 18 patients (11 %) at day 1, 12 patients (10 %) at day 3 and nine patients (18 %) at day 7. A total of 39 patients (21 %) developed a heel pressure ulcer during their hospital stay. The heel pressure ulcer categories varied between 1 and 3 at the ED and between 1 and 4 at the ward. There were no statistically significant differences regarding the patients’ vital signs, measured in the ambulance and on admittance to the ED, between patients who developed heel pressure ulcer and those who did not (Table 1).

**Table 1:** Vital signs in patients with and without pressure ulcer measured in the ambulance and at the ED^a^

Vital signs	Ambulance care		Emergency department	
	Pressure ulcer		Pressure ulcer	
	Yes Mean (SD)	No Mean (SD)	Yes Mean (SD)	No Mean (SD)
Respiratory rate	22 (6)	23 (6)	23 (16)	26 (27)
Heart rate	92 (26)	86 (20)	86 (24)	86 (20)
Reaction Level Scale	1 (0.4)	1 (0.5)	1 (0.5)	1 (0.5)
Pulse oximetry (%)	93 (6)	94 (7)	93(7)	95 (4)
Blood pressure (systolic) mmHg	140 (23)	146 (28)	139 (28)	147 (28)
Blood pressure (diastolic) mmHg	80 (17)	83 (19)	75 (18)	80 (18)
Temperature (C)	37 (1)	37 (0.8)	37 (7)	36 (0.9)

^a^No significant differences were detected

### Nursing actions

Of the included patients, 86 (60 %) were assessed as being at risk for developing pressure ulcer at the ED, 75 (53 %) at Day 1 at the ward, 37 (47 %) at Day 3 and 25 (64 %) at Day 7. Patients in the intervention group (*n* = 103) received a heel suspension device boot. Also, several nursing actions (use of pressure-reducing mattress, oral nutritional supplement, and turning schedule) to prevent pressure ulcers were taken at the ED and ward levels. However, not all patients with a heel pressure ulcer received pressure ulcer prevention actions (Table 2).

**Table 2:** Nursing actions for prevention of pressure ulcers at the emergency department and during hospital stay

Time points	Total number of patients with heel pressure ulcer *n*	Nursing actions	Heel pressure ulcer	
			Yes *n*	No *n*
Emergency department	15	Trolley (standard care for most patients)	15	148
		Bed	0	3
Hospital admission				
Day 1	18	Pressure-reducing mattress (bed)	10	93
		Turning schedule	9	41
		Heel suspension device boot^a^	6	88
		Oral nutritional supplements	10	36
Day 3	12	Pressure-reducing mattress (bed)	8	63
		Turning schedule	6	27
		Heel suspension device boot^a^	4	70
		Oral nutritional supplements	8	29
Day 7	9	Pressure-reducing mattress (bed)	5	27
		Turning schedule	2	17
		Heel suspension device boot	5	23
		Oral nutritional supplements	4	17

^a^Intervention group

(Due to internal dropout, the numbers do not add up to n.)

### Predictors for heel pressure ulcer

No variables (Additional file 1) assessed in the ambulance or at the ED predicted heel pressure ulcer during the hospital stay. However, at Day 1 at the ward, statistically significant differences were identified between patients who later developed heel pressure ulcer and those who did not, regarding the MNS sub-categories (mental condition (*n* = 144; mean rank = 55.8 vs mean rank = 76.5; *p* = 0.01), physical activity (*n* = 144; mean rank = 49.7 vs mean rank = 78.0; *p* = 0.001), mobility (*n* = 144; mean rank = 48.6 vs mean rank = 78.2; *p* < 0.000), and incontinence (*n* = 144; mean rank = 51.0 vs mean rank = 77.7; *p* = 0.002)), as well as total risk score (*n* = 142; mean rank = 50.3 vs mean rank = 76.7; *p* = 0.002). Also, at Day 1 at the ward, significantly fewer patients with a heel pressure ulcer or who developed heel pressure ulcer later during their hospital stay received an oral nutritional supplement compared to those with no heel pressure ulcer (*n* = 142; *n* = 17 vs *n* = 30; *p* < 0.000). Statistically significant differences could not be noted regarding other preventive actions, such as using a pressure-reducing mattress and a turning schedule.

## Discussion

Findings from the present study revealed that several of the included patients were assessed as being at risk for developing pressure ulcer, and that heel pressure ulcer develops across the care delivery chain (i.e pre-hospital and emergency care level, and further at the hospital ward). However, all patients with a hospital-acquired heel pressure ulcer did not receive adequate pressure ulcer prevention actions during their hospital stay, even though national guidelines [10] have been introduced.

This patient group can be commonly seen in different healthcare settings and is often not prioritized in emergency care settings, which may lead to prolonged waiting times and an increased risk for developing pressure ulcer. Some of the included patients had an ambulance transportation time of longer than an hour, which means there was additional time during which they were in an immobilized state on a stretcher. Waiting at the ED on a trolley may lead to increased risk for developing pressure ulcer. Research has informed us that cellular damage and thus the potential development of pressure ulcers can occur within a few minutes [16].

Our findings identified that a number of patients developed heel pressure ulcers at a time between inclusion in the ambulance and during their time at the ED. However, we do not know the exact time-point and the cause of the pressure ulcer. One possible explanation might be the ambulance staff’s knowledge level about pressure ulcer and how to perform the skin inspection. Another explanation could be the patient’s immobilized state on a stretcher. It would be valuable to further study the nursing actions carried out in the ambulance to identify how to improve the preventive care in the ambulance service. Reducing pressure ulcer prevalence should be used as a quality indicator even in the pre-hospital care. Early identification of pressure ulcer or information about the result from the skin inspection could then be reported to the staff in the ED to continue the preventive work. This should be an applicable intervention in the pre-hospital setting. The characteristics of the included patients show that this is a vulnerable patient group, often with reduced mobility, higher temperature and lower oxygen saturation. These factors are all crucial to consider in pressure ulcer prevention [10]. But in our study, neither vital signs nor MNS scores, assessed at the ED, were identified as predictors for developing heel pressure ulcer later in the care delivery chain. The reason for this could be that vital signs and MNS scores in the initial stage can be difficult to assess, and can rapidly change to a more normal level after treatment at the ED; this sometimes leads to a situation in which the patient is discharged to home. At the time of the study, risk assessment using a validated instrument was not routinely conducted at the included EDs. Subsequently, limited knowledge about performing adequate MNS assessment might have affected the assessment scores. However, it could also be that initial skin inspection in the ambulance and at the ED is the most appropriate level of preventive action in the early stage of the care delivery chain. It may also be the most effective way to use emergency resources. However, the result of the skin inspection needs to be communicated to the receiving ward [25].

On the other hand, risk assessment using MNS seems to be of great importance at the ward level. Also, when patients are identified as being at risk, appropriate actions are necessary to prevent pressure ulcers. In our study, findings showed that actions were taken, but without considering whether or not the patients were assessed as being at risk for developing heel pressure ulcer. But there were also situations in which no action was taken, even though patients were identified as being at risk for developing pressure ulcer. Risk assessments need to be conducted regularly during the hospital stay, and appropriate actions need to be taken, documented and evaluated.

With the findings from this study, the arguments for early implementation of appropriate preventive actions are strengthened. The challenge is identifying patients with an increased probability of pressure ulcer development within the ambulance care and emergency care settings, where pressure ulcer prevention has not been prioritized. In the updated international guidelines the general recommendation for structured risk assessment and skin assessment is that it should be implemented at first contact with the health professionals [10]. For preventing pressure ulcer, research highlights that skin inspection, mobilization, and circulation are the key indicators [10]. It is important to have knowledge of the mechanisms that lead to tissue damage even at the initial stage of the patient’s care process.

Pressure ulcer prevalence is still at an unacceptably high level in many healthcare settings. However, this knowledge, as well as quality indicators, is information mostly used by managers, researchers and policy-makers. Research informs us that, despite the development of evidence-based clinical guidelines, they are not always used in clinical practice [8]. Most of the clinical guidelines are quite comprehensive, and have been developed for in-hospital care [10, 22] and do not address the context challenges of the care provided, for example, in the ambulance or at the ED. Annual national pressure ulcer prevalence studies are conducted, but exclude the ambulance and the emergency department settings. Neglecting to address these settings may signal that pressure ulcer prevention is of no importance in these settings. Our study shows the opposite: pressure ulcer prevention is important also in the early stage of the care delivery chain.

The present study, originating from a RCT, highlighted the importance of undertaking research in complex acute care situations. Although the number of participants was limited, the findings showed differences in pressure ulcer prevention across the care delivery chain. Research studying pressure ulcer prevention and predictors for pressure ulcer often focuses on specific contexts such as hospital wards and nursing homes, where the situation can be much more controlled compared to acute care situations. However, to improve nursing care and decrease hospital-acquired pressure ulcer, further research investigating preventive actions in different contexts, including pre-hospital and emergency care settings, is warranted.

Findings from this study can be used by clinicians to guide nursing care, i.e. when to take action, and what action to be taken at what time. However, these findings are also important to managers, for the evaluation of the quality of care and for comparisons.

### Limitations

This study was not without challenges and limitations, mainly related to the data collection method. Several different kinds of healthcare settings and healthcare professionals were involved, potentially leading to variety in the assessments. Data about preventive nursing actions in the ambulance, besides application of the heel suspension device boot, were not retrieved. There were no standardized routines for pressure ulcer prevention in the ambulance care. However, individual actions would have been important to address. Also, it was not possible to retrieve information about the patients’ total time at the ED. Differences in waiting times may have affected the incidence of heel pressure ulcer. Due to resource limitations, it was not feasible to monitor every step in the data collection process for each patient. On the other hand, the inclusion of different settings is one of this study’s strengths, as few studies go beyond pressure ulcer prevention across the care delivery chain; to our knowledge, this is one of the first. More and larger studies are needed to confirm these findings. Also, studies investigating other types of pressure ulcer than heel pressure ulcer would be of interest.

## Conclusion

Pressure ulcer prevention should be incorporated into the patient care from the very beginning of the care delivery chain. However, these actions should be context-tailored, with skin inspection appearing to be appropriate for preventing pressure ulcer in the ambulance and at the emergency department. On the other hand, carrying out risk assessment is important at the ward level; this would also be an appropriate level for the use of healthcare resources.

## Additional file

Additional file 1: Table S1. Variables and statistical analyses used in the data analysis. (DOCX 19 kb)
