# Supplementary material for: Heel pressure ulcer, prevention and predictors during the care delivery chain – when and where to take action? A descriptive and explorative study
Source: Scand J Trauma Resusc Emerg Med. 2016 Nov 14;24:134. doi: 10.1186/s13049-016-0326-0 (PMC5109774; doi:10.1186/s13049-016-0326-0)
Supplement: Additional file 1: Table S1. — Variables and statistical analyses used in the data analysis. (DOCX 19 kb) [file 13049_2016_326_MOESM1_ESM.docx]

Table 1. Variables and statistical analyses used in the secondary analysis.

| Variable | Statistical analyses | | | |
| --- | --- | --- | --- | --- |
|  | Descriptive statistics | Chi-2 test | t-test | Mann-Whitney U-test |
| **Outcome** | | | | |
| Heel pressure ulcer | x |  |  |  |
| **Patient characteristics** | | | | |
| Age | x |  | x |  |
| Gender | x | x |  |  |
| Transportation time (ambulance) | x |  | x |  |
| Vital signs (respiratory rate, systolic blood pressure, diastolic blood pressure, heart rate, Reaction Level Scale, temperature control and pulse oximetry) (ambulance and ED) | x |  | x |  |
| **Pressure ulcer prevention** (ED, ward) | | | | |
| MNS: Mental condition |  |  |  | x |
| MNS: Physical activity |  |  |  | x |
| MNS: Mobility |  |  |  | x |
| MNS: Food intake |  |  |  | x |
| MNS: Fluid intake |  |  |  | x |
| MNS: Incontinence |  |  |  | x |
| MNS: Physical condition |  |  |  | x |
| Risk or no risk for developing pressure ulcer | x | x |  |  |
| Total risk score | x |  |  | x |
| Skin inspection | x | x |  |  |
| Heel pressure ulcer category | x |  |  |  |
| Heel suspension device boot | x | x |  |  |
| Pressure-reducing mattress | x | x |  |  |
| Oral nutritional supplement | x | x |  | x |
| Turning schedule | x | x |  |  |

^ED = Emergency department^

^MNS= Modified Norton Scale^
